# Usefulness of ultrasound in diagnosing constipation in children: a narrative review

**DOI:** 10.3389/fped.2026.1813027

**Published:** 2026-05-29

**Authors:** Katarzyna Bąk-Drabik, Martyna Laskowska, Katarzyna Głuszko, Marta Drabik, Giovanni Maconi

**Affiliations:** 1 Department of Pediatrics, Faculty of Medical Sciences in Zabrze, Medical University of Silesia in Katowice, Zabrze, Poland; 2 Students’ Scientific Association at the Department of Pediatrics, Faculty of Medical Sciences in Zabrze, Medical University of Silesia in Katowice, Zabrze, Poland; 3 Gastroenterology Unit—Department of Biomedical and Clinical Science - ‘L.Sacco’ University Hospital, University of Milan, Milan, Italy

**Keywords:** children, constipation, fecal load, perianal ultrasound, transabdominal intestinal ultrasound

## Abstract

Chronic constipation in children is a significant problem not only for the child but also for the family and caregivers in institutional settings. Constipation is a common clinical symptom, accounting for nearly one-third of consultations in pediatric gastroenterology clinics. In the vast majority of cases, the cause is functional constipation, whose pathogenesis is complex but usually benign. The greatest challenge lies in identifying organic causes within this patient group, for which ultrasound is particularly well suited as a non-invasive tool for exclusion. Transabdominal intestinal ultrasound is an ideal diagnostic tool due to its non-invasive, cost-effective, and repeatable nature, and its ability to be performed during routine outpatient visits. It enables objective assessment of fecal retention by measuring rectal diameter (RD), rectal wall thickness, and degree of colonic filling. In addition, ultrasound may be useful for assessing stool consistency and colonic transit. Perianal ultrasonography is a complementary method to transabdominal intestinal ultrasound, enabling a more detailed evaluation of anal canal structures. Ultrasound may therefore represent a valuable adjunct in the diagnosis and management of pediatric constipation, although further standardization of methodology and diagnostic criteria is required.

## 1 Introduction

Constipation is a common symptom worldwide, characterized by considerable variation in its definition (1). Most simply definition, it is characterized by excessively long intervals between bowel movements (fewer than two defecations per week) and/or the passage of stools that require considerable straining (2). Constipation accounts for around 3% of all referrals to general pediatric outpatient clinics and 11%–25% of visits to pediatric gastroenterologists (3, 4).

The most important thing, because of significantly different treatment strategies, is to distinguish between organic and functional constipation (FC). The latter is a clinical diagnosis based on the Rome IV criteria (2), and its pooled prevalence is estimated at 8.17% among children aged 0–4 years and 11.39% among children aged 4–18 years (5). Organic causes of constipation in the pediatric population are relatively rare. Although the exact prevalence has not been clearly established, it is estimated to account for approximately 5% of cases (6).

Ultrasound (US) is an imaging method for examining the internal body anatomy, utilising the generation and reception of ultrasonic waves. Because the US is safe, compact, inexpensive, radiation-free, and operates in real time, it is widely used in medical practice, including pediatrics (7). The US apparatus consists of a console and a transducer. The most commonly used US probes are linear, convex, microconvex, and phased arrays. In pediatrics, microconvex, or convex linear arrays (CAs) with a smaller radius of curvature (ROC), ordinarily operating in the frequency range 3–11 MHz, are widely used (7). In the increasing use of transabdominal ultrasonography for diagnosing constipation in children linear (7–15 MHz), convex (3.5–6 MHz), or mini-convex (4–13 MHz) probes are applied (8–12). The Polish Society for Pediatric Gastroenterology, Hepatology, and Nutrition agrees that abdominal US with visualization of the rectum through a filled bladder can be a helpful diagnostic tool for constipation (6). Despite valuable research on this issue, the European Society for Pediatric Gastroenterology, Hepatology and Nutrition (ESPGHAN) and the North American Society for Pediatric Gastroenterology, Hepatology and Nutrition (NASPGHAN) do not support the routine use of transabdominal rectal US for diagnosing functional constipation (13).

## 2 Methodology

A narrative literature review was conducted to evaluate the role of ultrasound (US) in the diagnosis and assessment of constipation, with a particular focus on the pediatric population. PubMed/MEDLINE and Google Scholar were searched, and results were limited to relevant papers published in English.

The search strategy was based on a Boolean search string: (coprostasis OR constipation OR “fecal loading” OR “rectal diameter”) AND (children OR pediatric) AND (ultrasound OR ultrasonography OR sonography OR “perianal ultrasonography”), covering the period from 1990 to February 2026. This search yielded 860 records, which were screened for relevance in the field (see below).

Additionally, further relevant studies were identified through manual screening of reference lists, clinical guidelines, and position papers. Given the narrative nature of this review, study selection was based on clinical relevance, methodological quality, and contribution to the topic. Studies were included if they: i) evaluated the use of US in the assessment or diagnosis of constipation; ii) reported US parameters [e.g., rectal diameter (RD), fecal loading, or qualitative findings]; iii) involved pediatric populations or provided clinically relevant data applicable to children; iv) included adult populations only when pediatric data were not available and the studies provided clinically relevant information; v) were original research articles, randomized controlled trials, observational studies, or clinical guidelines published in English. All identified records were screened based on titles and abstracts. Full-text articles were subsequently assessed for eligibility according to predefined inclusion criteria. Both studies demonstrating US's usefulness and those reporting limited or no diagnostic value were considered to provide a balanced overview of the available evidence.

Studies were excluded if they were non-English, lacked full-text access, did not assess ultrasound in constipation, did not report relevant ultrasonographic parameters, or were non-original (e.g., reviews, editorials, conference abstracts) or preclinical studies.

Data extracted from the included studies comprised: i) study design and population (including age group and sample size); ii) US technique and type of transducer; iii) measured parameters (e.g., RD, thickening rectal wall, colonic filling, gastrointestinal luminal content, organic causes of constipation); iv) main outcomes and conclusions.

## 3 Functional constipation (FC)

The diagnosis of FC is based on the Rome IV criteria. It relies on the patient's history and physical examination, including the frequency and consistency of bowel movements, the presence of stool withholding behaviors, painful defecation, fecal incontinence, and the occasional passage of large stools. Importantly, the diagnosis requires excluding organic causes, as functional constipation is defined by the absence of underlying structural, metabolic, endocrine, or neurological disorders (4). According to the Rome IV criteria, routine use of abdominal US is not recommended for diagnosis and should be reserved for patients presenting with alarm features suggestive of an underlying organic disorder (4). US assessment may assist in managing FC. Notably, subjective symptom descriptions are often unreliable in infants and many older children. Therefore, clinicians frequently request additional imaging studies, such as abdominal radiographs or US, during the diagnostic or therapeutic process. US enables evaluation of, among others, fecal loading, fecal properties, the thickness of the rectal wall, fecal filling of the entire large intestine, and the exclusion of anatomical and organic abnormalities in the abdominal cavity (14–18).

### 3.1 Assessment of fecal loading

A commonly cited definition of luminal distension refers to a small-bowel diameter greater than 25 mm and a colonic diameter greater than 50 mm (19). The diameter of the bowel varies according to site, fasting/feeding state, and bowel function. On US, fecal loading refers to increased intraluminal stool with associated bowel dilatation and typical echogenic patterns (dark solid mass with posterior shadowing), assessed qualitatively rather than by strict diameter thresholds (19).

**Figure 1:**
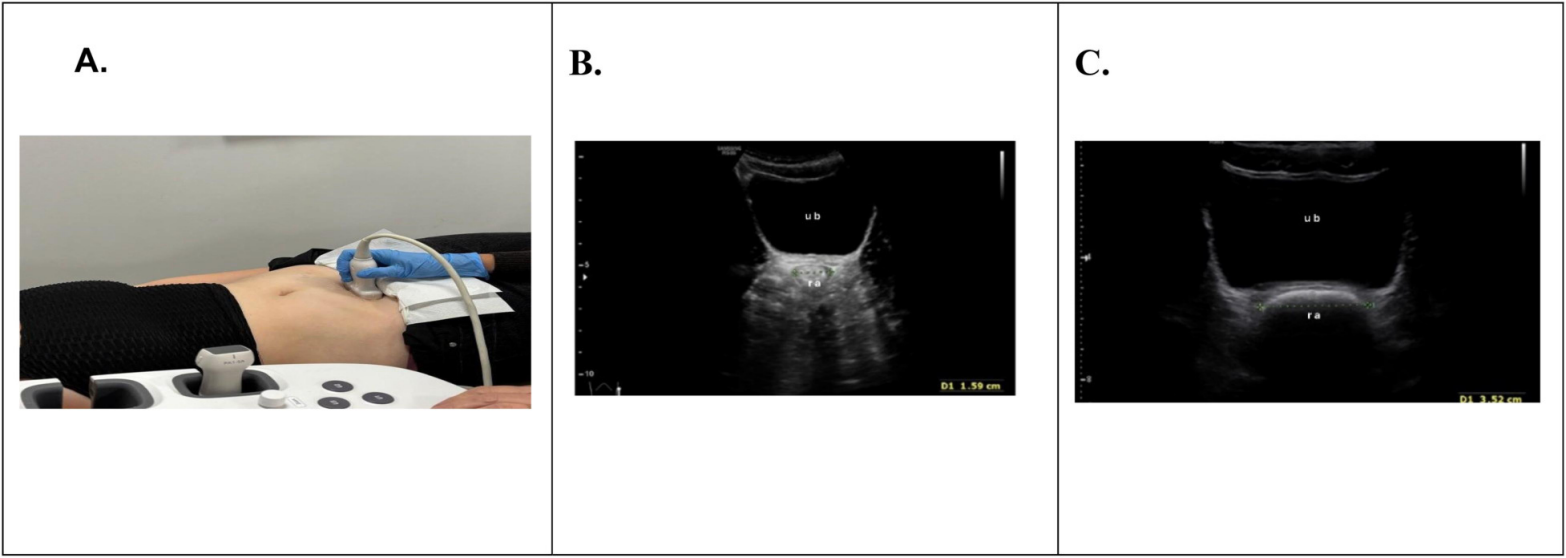
**(A)** proper positioning of the US transducer for rectal assessment. **(B)** Transverse view of an empty rectum. **(C)** Transverse view of an enlarged rectal ampulla in a child with faecal retention, urinary bladder (u b), rectal ampulla (r a).

In the vast majority of studies, the degree of fecal loading has been assessed by measuring the transverse (RD). Accurate measurement of the RD is a key element of the assessment process. The transverse (RD) should be measured using US with the child's bladder moderately filled to provide an acoustic window (12). A sector or curved-array probe (typically 3.5–7.5 MHz) is positioned on the lower abdomen, approximately 2 cm above the symphysis, and angled downward by approximately 10–15° from the transverse plane. The rectum must be clearly distinguished from the sigmoid colon, and the largest transversal diameter is recorded (Figure 1) (9, 12, 20). Hatori et al. (12) measured RD using transabdominal ultrasonography in three planes: above the symphysis, below the ischial spine, and at the bladder neck. Comparison of these approaches demonstrated that the measurement obtained above the symphysis most accurately reflected fecal retention and showed the strongest diagnostic correlation, making it the most suitable projection for clinical use. Figure 1. (A) illustrates the appropriate positioning of the US transducer for accurate rectal assessment. (B) shows US images from a child without constipation (C) shows a child with constipation.

Evidence shows that RD can be used to detect fecal retention in children (presented in detail in Table 1) (8–23).

**Table 1:** A summary of available original articles addressing the assessment of the width of the rectal ampulla in children.

Study	Study population	Results and conclusion
(15)	50 children with constipation (25 boys), mean age 7 years, 50 healthy controls (31 boys), mean age 6 years.	RD was higher in constipated vs. healthy children [35 mm [IQR 32–40] vs. 23 mm (18–25), *p* < 0.001]. A cut-off >3 cm was suggested for children >4 years. After 3 months, RD decreased to 26 mm (20–28, *p* < 0.001).
(16)	50 children with abdominal pain: 32 constipated (10 boys; mean age 9.5 years) and 18 non-constipated (8 boys; mean age 10.5 years).	The RD was greater in constipated children vs healthy. (43 ± 13,5 mm vs. 28,5 ± 11.6 mm; *p* < 0.001. A RD cut off of 3.8 cm or greater correlated with the diagnoses of constipation (*p* < 0.001).
(22)	34 children with constipation (22 boys), mean age 5.6 years, 31 healthy children (14 boys), mean age 5.4 years	The RD was greater in constipated vs healthy (34.9 ± 14.1 mm vs. 24.2 ± 7.1 mm; *p* = 0.001. 23 (67%) had a RD larger than 30 mm.
(17)	120 children with costipation (72 boys), aged 1.6–17.9 years, mean age 6.25 years, 105 healthy controls	The RD differed between constipated and healthy cases (43.060 ± 9.68) vs. 31.83 ± 8.24 (*p* < 0.001. Additionally, stool retention was assessed using the rectopelvic ratio (the relationship between the RD and the transverse pelvic width). The numerical values of this parameter differed significantly in all age patients (*p* < 0.001).
(8)	30 children with constipation (18 boys) aged 1,5–9 years. 46 healthy controls.	The RD was greater in children with constipation vs healthy. (31.72 ± 9.63 mm vs. 19.85 ± 4.37 mm; *p* = 0.001
(11)	35 children with constipation (mean age, 6.8 ± 2.9 years), 31 control cases (mean age, 8.4 ± 3.8 years)	The mean RD was thicker in the constipated group (3.02 ± 1.04 cm) than in the control (1.98 ± 0.64 cm) (*P* < .001). The cutoff point of RD for a diagnosis of constipation was determined as 2.44 cm
(12)	51 children with constipation aged 4–12 years 24 healthy controls	The RD was significantly larger in constipated children (42.1 ± 15.4 mm) than in healthy patients (21.4 ± 6.0 mm; *p* < 0.001). Significant reduction in RD (mean 26.9 ± 5.6 mm, *p* < 0.001) was observed after 4 weeks of laxative treatment.
(23)	140 children with constipation (62 boys), mean age (8.1 ± 5.2) years, 164 healthy controls (68 boys), mean age 8.5 ± 5.2 years	RD was significantly larger in children with constipation aged 6.1–12 years than in healthy controls (33.41 ± 7.21 vs 27.55 ± 7.11; *p* < 0.001), measured above the pubic symphysis, below the ischial spine, and at the bladder neck.
(13)	100 children (53 boys) (median age, 5.0 years), 80 cases with functional constipation and 20 without constipation,	RD was greater at all measurement points—above the symphysis, below the ischial spine, and at the bladder neck—in children with fecal impaction (35.2 vs 20.9 mm; 35.7 vs 24.0 mm; 19.4 vs 8.7 mm; *P* < 0.0001); the cut-off above the symphysis was 27 mm.

According to 2024 meta-analysis and systematic review included 14 methodologically robust studies, an RD cut-off of 31 mm demonstrated high diagnostic performance when the Rome criteria were used as the diagnostic reference standard for FC (8). However, this cut-off threshold of 31 mm for RD should be carefully applied to other ages than in toddlers and young children.

**Figure 2:**
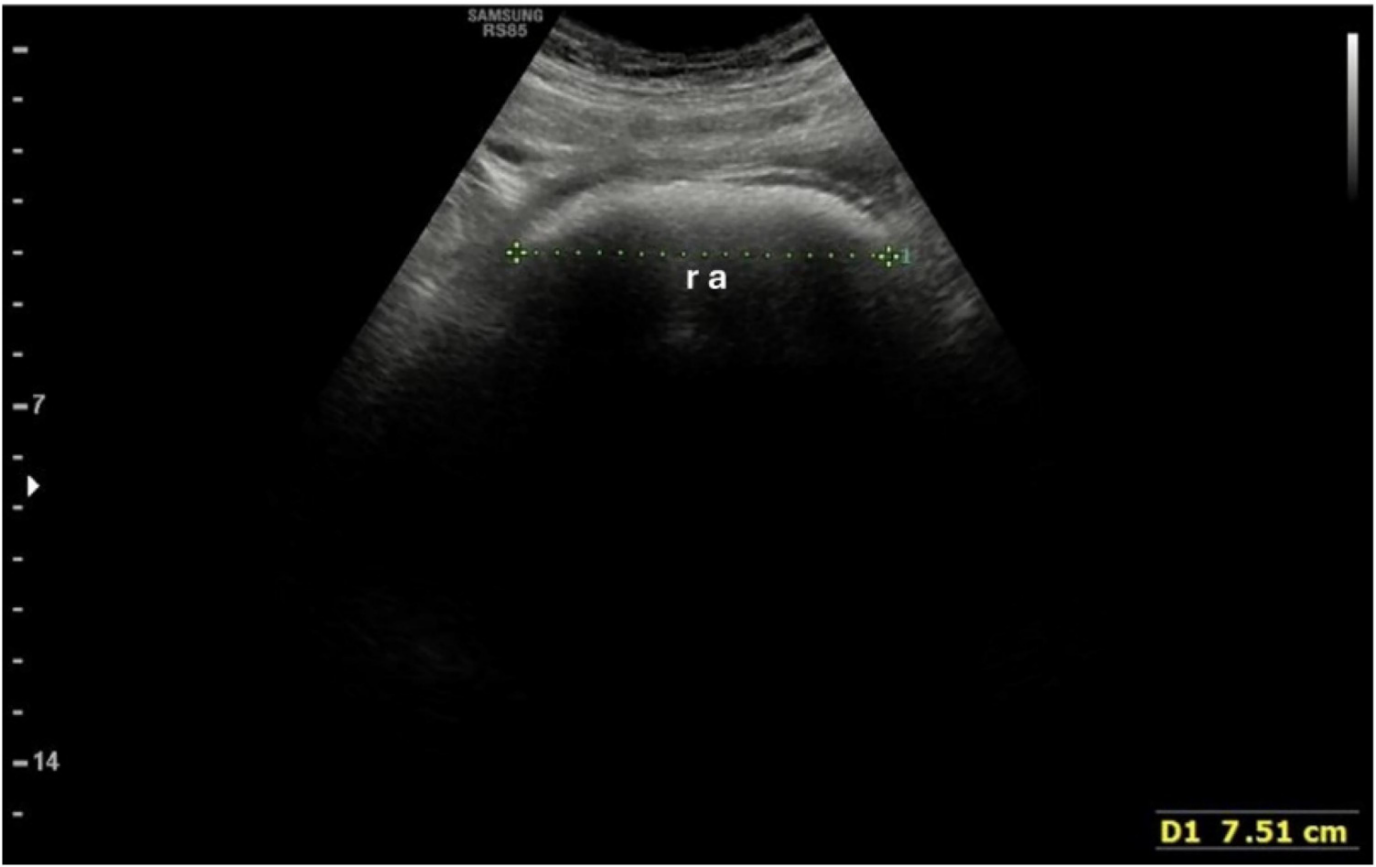
An 4,5 -year-old boy with functional chronic constipation. The transverse diameter of the rectal ampulla (r a) measures 7.5 cm.

In the Expert Position Statement published in 2025 (24), 43 statements were deemed appropriate after two rounds of voting by an international panel comprising 14 experts in adult and pediatric gastroenterology and one radiologist experienced in gastrointestinal imaging. A RD of 30 mm was considered appropriate to define fecal loading in children. No recommendation was made for adult patients due to the lack of data. This document confirms the clinical applicability of ultrasonographic RD measurement as a valuable, non-invasive diagnostic tool for evaluating FC in children. Figure 2 shows rectal ampulla dilatation with hard stool.

#### 3.1.1 Assessment of rectal ampulla dilatation during anti-constipation treatment

It should be noted that rectal ampulla dilatation exceeding 30 mm may persist for a considerable period of time, even after appropriate and effective treatment and the passage of soft stools (25).

This phenomenon is related to impaired rectal contractility resulting from chronic fecal retention. As Di Pace et al. (25) emphasized, clinical improvement in children with chronic constipation often precedes normalization of the RD; therefore, medical therapy should be continued until the rectal ampulla returns to normal size on US, rather than discontinued once regular bowel habits are achieved.

**Figure 3:**
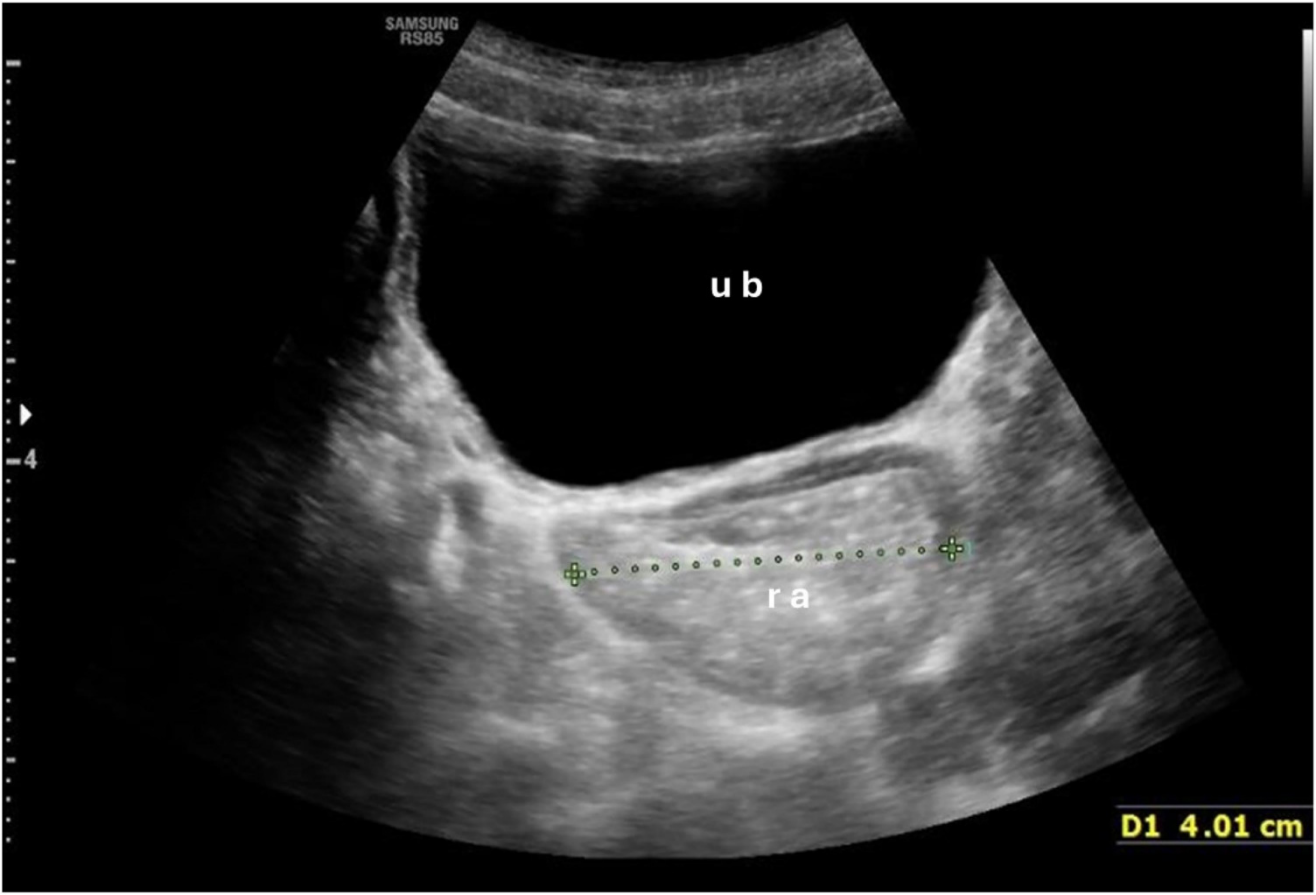
Dilated rectum with normal stool in treatment-resistant constipation (2.5-year-old boy; ub: urinary bladder, (r a: rectal ampulla).

Figure 3 shows a persistently dilated rectal ampulla despite appropriate medical treatment, normalization of bowel habits and improvement of clinical symptoms.

Optimal timing for assessing RD after the initiation of anti-constipation therapy is important. Our experience suggests that a reduction in RD can be expected after approximately 4 weeks of treatment. This observation is supported by a study that demonstrated a significant decrease in transverse RD after 4 weeks of therapy; however, the RD remained significantly larger than that observed in healthy children (39.6 ± 8.2 mm vs. 26.9 ± 5.6 mm; *p* < 0.001) (11).

Di Pace et al. (25) proposed a monthly US follow-up to provide feedback to children and their caregivers and offer guidance on the appropriate timing for tapering or discontinuing laxative therapy. Clinical guidelines do not define the optimal duration of patient follow-up (10, 11). Personal experience supports monthly monitoring at the beginning of therapy, which may later be extended to every 3 months and subsequently to every 6 months. The US findings may help distinguish between the two main types of FC—colonic inertia, characterized by delayed colonic transit, and outlet obstruction, resulting in stool retention in the rectum—as their management strategies differ significantly.

Patients with rectal dilatation may benefit from fecal disimpaction using enemas. However, Di Pace et al. (25) suggested that enemas should not be routinely added to standard laxative therapy as a preventive measure in patients with a RD ≤ 3.0 cm, even when bowel movement frequency is ≤3 episodes per week. Thus, ultrasonography is a valuable tool in clinical decision-making.constip.

**Figure 4:**
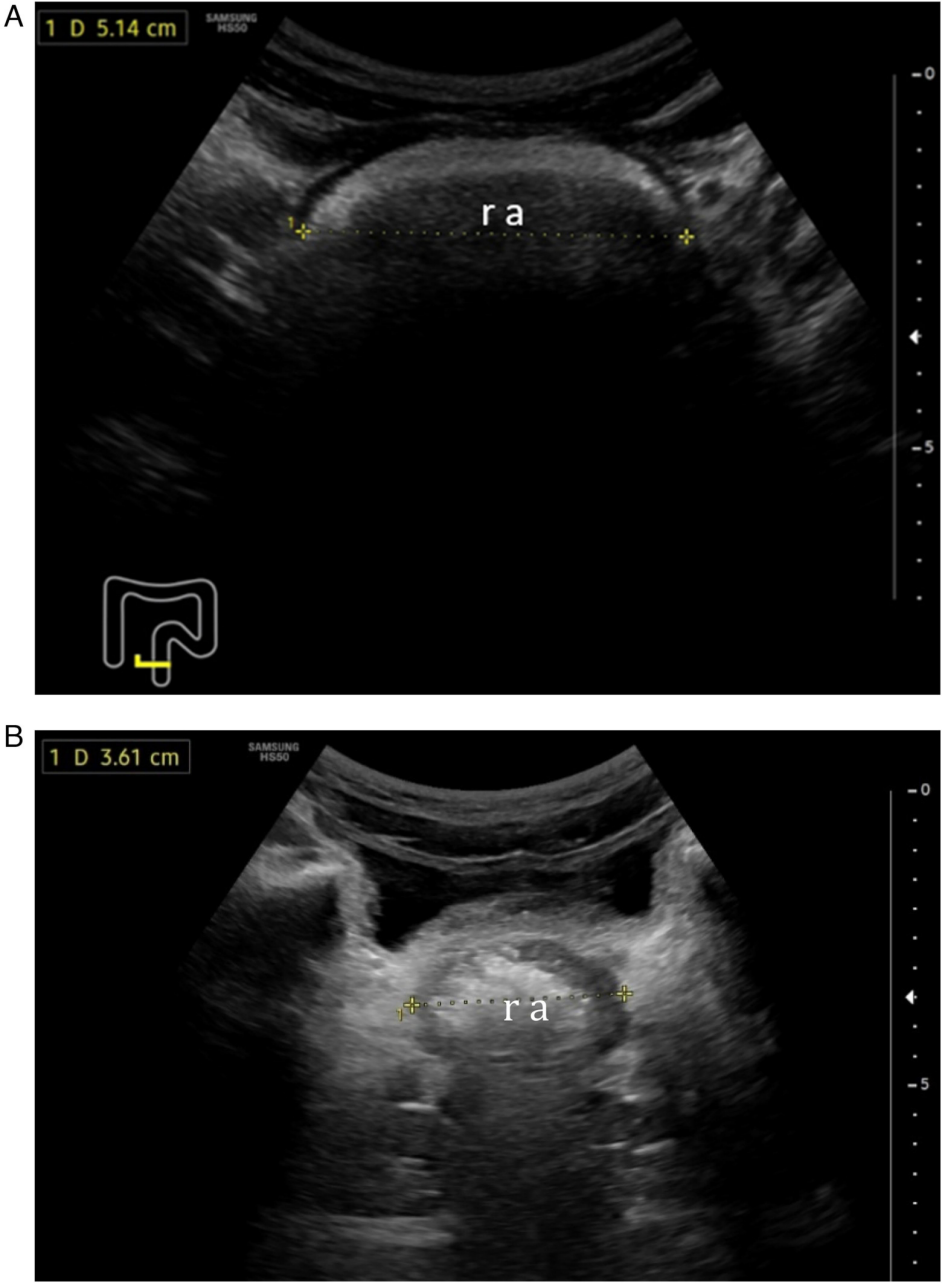
**(A)** An 8-year-old girl with functional chronic constipation. The transverse rectal diameter (r a) measures 5.14 cm before treatment. **(B)** An 8-year-old girl with functional chronic constipation. The transverse rectal diameter measures 3.61 cm after 2,5 months of treatment. (r a: rectal ampulla).

Figure 4 shows the difference between the rectal ampulla before and after anti-constipation treatment in the same patient.

Pop et al. (22) demonstrated that rectal dilatation was not related to the duration of constipation. This finding raises the important question of whether the primary pathophysiological problem might be an altered sensory and motor function of the rectum. Such patients may require specialized US monitoring for both diagnostic assessment and follow-up during constipation treatment.

#### 3.1.2 Additional considerations in the sonographic assessment of fecal loading

While most studies have evaluated transverse RD, some have explored additional sonographic parameters assessing fecal loading, such as the retropelvic ratio, which is the ratio of the transverse rectal to transverse pelvic width (17). The other new tool was the US scoring system, designed to assess the fecal loading. The authors assessed the degree of rectal ampulla filling from the retrobladder region up to the level above the umbilicus, in some cases, the upper edge could not be visualized. The second evaluated parameter was the effect of the fecal mass on the urinary bladder (26).

The typical site for assessing RD was the suprapubic region; however, some authors (12) used additional measurements above the symphysis pubis, such as under the ischial spine and at the bladder neck, and reported that measurements obtained above the symphysis pubis showed the strongest correlation with fecal retention. The cutoff for RD above the symphysis to identify fecal retention was 27 mm, with high sensitivity (95.5%) and specificity (94.1%).

Another study also compared the RD between full and empty bladders. It was found that assessing RD with the bladder empty was more informative and indicated that rectal wall thickness was greater in children with constipation (27). Contrary to the study by Doniger et al. (16), RD measurement was not affected by the degree of bladder fullness.

A digital rectal examination (DRE) is also used to assess the presence of feces in the rectum. Still, this examination is associated with discomfort and is not preferred by either children or parents (13). Radiologic imaging was historically used to assess fecal loading; however, current ESPGHAN and NASPGHAN guidelines conclude that available evidence does not support the use of abdominal radiography for the diagnosis of functional constipation (13).

### 3.2 Assessment of the rectal wall thickness

Rectal wall thickness provides complementary information on rectal distension. Increased wall thickness may indicate chronic stool retention. Several studies have demonstrated significant differences in rectal wall thickness between children with constipation and healthy children (10, 22, 23). However, most of them assessed only the anterior rectal wall without explaining the apparent asymmetry (10, 22). In one study that compared mean wall thickness, there was no difference between children with constipation and controls (28). The discrepancies in the results likely reflect measurement artifacts, including oblique measurements due to the posterior inclination and compression by the urinary bladder. Moreover, during US examination, the anterior wall is often the only part visualized, limiting accurate assessment.

**Figure 5:**
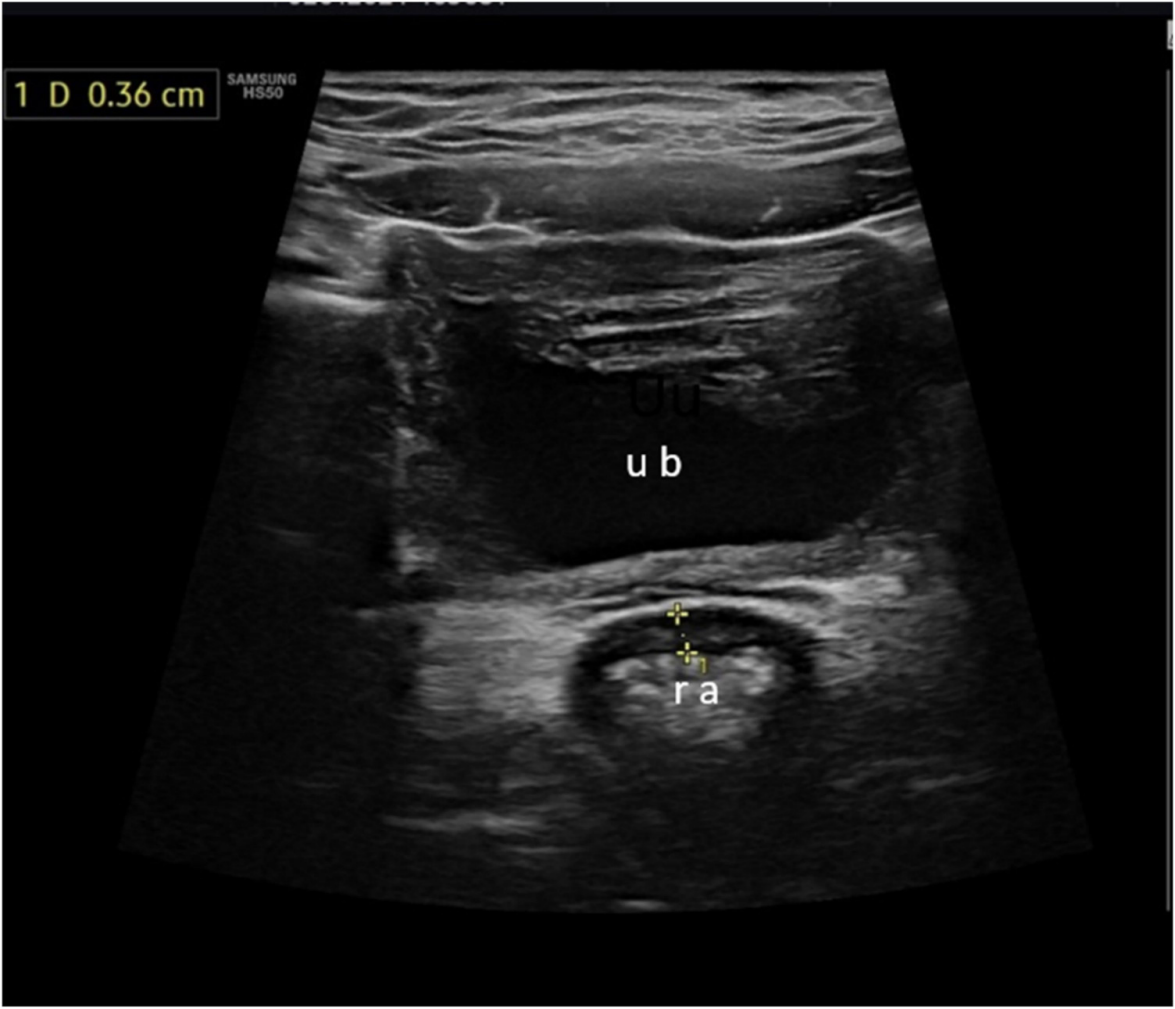
Empty rectum with wall thickening (4.3 mm) in treatment-resistant constipation (6-year-old boy ub: urinary bladder, (r a: rectal ampulla).

Histopathological studies suggest that rectal wall thickening may reflect hypertrophy and remodeling of the muscularis propria in response to chronic fecal retention (29, 30). However, US cannot determine which specific layer is most responsible for this finding, and further studies are required (Figure 5). Detailed findings of studies evaluating rectal wall thickness are summarized in Table 2.

**Table 2:** A summary of available original articles addressing the assessment of rectal wall thickness.

Study	Study population	Parameter	Results and conclusion	Significance
(23)	304 children (140 with constipation, 164 controls), age groups ≤3, 3.1–6, 6.1–12, >12 years	Anterior rectal wall thickness	Higher thickness in children with constipation; correlation with duration (r = 0.40)	*p* < 0.05; *p* = 0.000
(28)	100 children with constipation vs 100 controls (age 2–18 years)	Rectal wall thickness	Higher mean thickness (1.68 vs 1.29 mm)	Not significant (*p* = 0.94)
(10, 22)	Children with constipation vs controls	Anterior rectal wall thickness	Increased thickness in constipation	*p* < 0.01
(29, 30)	Megarectum studies	Rectal wall structure	Hypertrophy and remodeling of the muscularis propria	Supports adaptive changes due to chronic fecal retention

## 4 Ultrasound assessment of the character of gastrointestinal luminal content

Sonographic assessment of fecal properties is feasible and can provide useful information for clinical practice, although data in the pediatric population are scarce and largely derived from adult studies.

**Figure 6:**
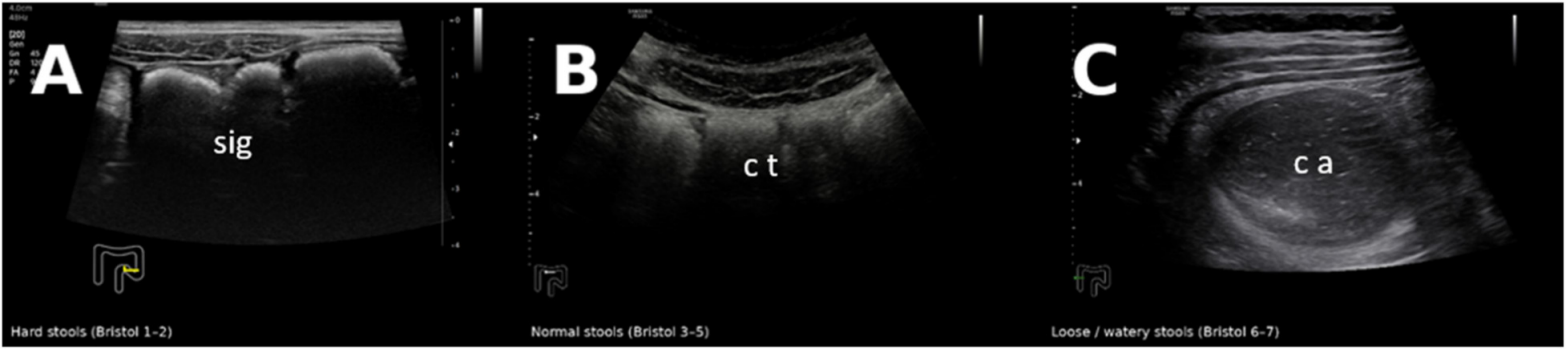
Different degrees of stool hardness corresponding to the Bristol Stool Scale: **(A)** hard stools (Bristol 1–2) in the sigmoid colon (sig), **(B)** normal stools (Bristol 3–5) in the transverse colon (ct), and **(C)** loose/watery stools (Bristol 6–7) in the ascending colon (ca).

A Japanese study conducted in an elderly population (29 patients, 129 US examinations) introduced a transgluteal cleft approach for evaluating fecal impaction and characteristics (classification of stool consistency according to the Bristol Stool Scale) in the lower rectum. US patterns included rock-like echogenic areas indicating hard stools (Bristol 1–2), irregular cotton candy-like areas indicating normal stools (Bristol 3–5), and flat mousse-like areas corresponding to muddy or watery stools (Bristol 6–7) (Figure 6).

Linear echogenic lines with a clearly visualized anterior rectal wall indicated the absence of stool. While the transabdominal approach remains the standard in children, similar technical limitations to those seen in adults—such as bowel gas and insufficient bladder filling—may occur (31).

Another adult study (32) compared ultrasonographic with computed tomography results, stating that US can be used to assess the hardness or softness of fecal loading. They authors concluded that US images, which are connected with fecal loading, are: the crescent-shaped acoustic shadows (the bright reflection of the anterior surface) with haustrations and deep acoustic shadowing, indicating the loading of hard feces in the colon, strong high echoes of the colonic lumen with no haustrations and a flat outer surface indicating abundant gas in the colon lumen and weak high echoes of the colonic lumen (the weak high echoes correspond to a higher colon-gas ratio, indicating softer stool consistency).

Tanaka et al. (33), in their study of elderly patients, assessed the presence of acoustic shadow, reflection, and haustra form. They classified four patterns of fecal distribution changes in the colon: no acoustic shadow, weak reflection with acoustic shadow, moderate reflection with acoustic shadow, strong reflection with acoustic shadow, and haustra-shaped strong reflection with acoustic shadow. Similarly, they assessed three patterns of fecal distribution changes in the rectum: no acoustic shadow, a half-moon-shaped moderate reflection with an acoustic shadow, and a crescent-shaped strong reflection with an acoustic shadow.

**Figure 7:**
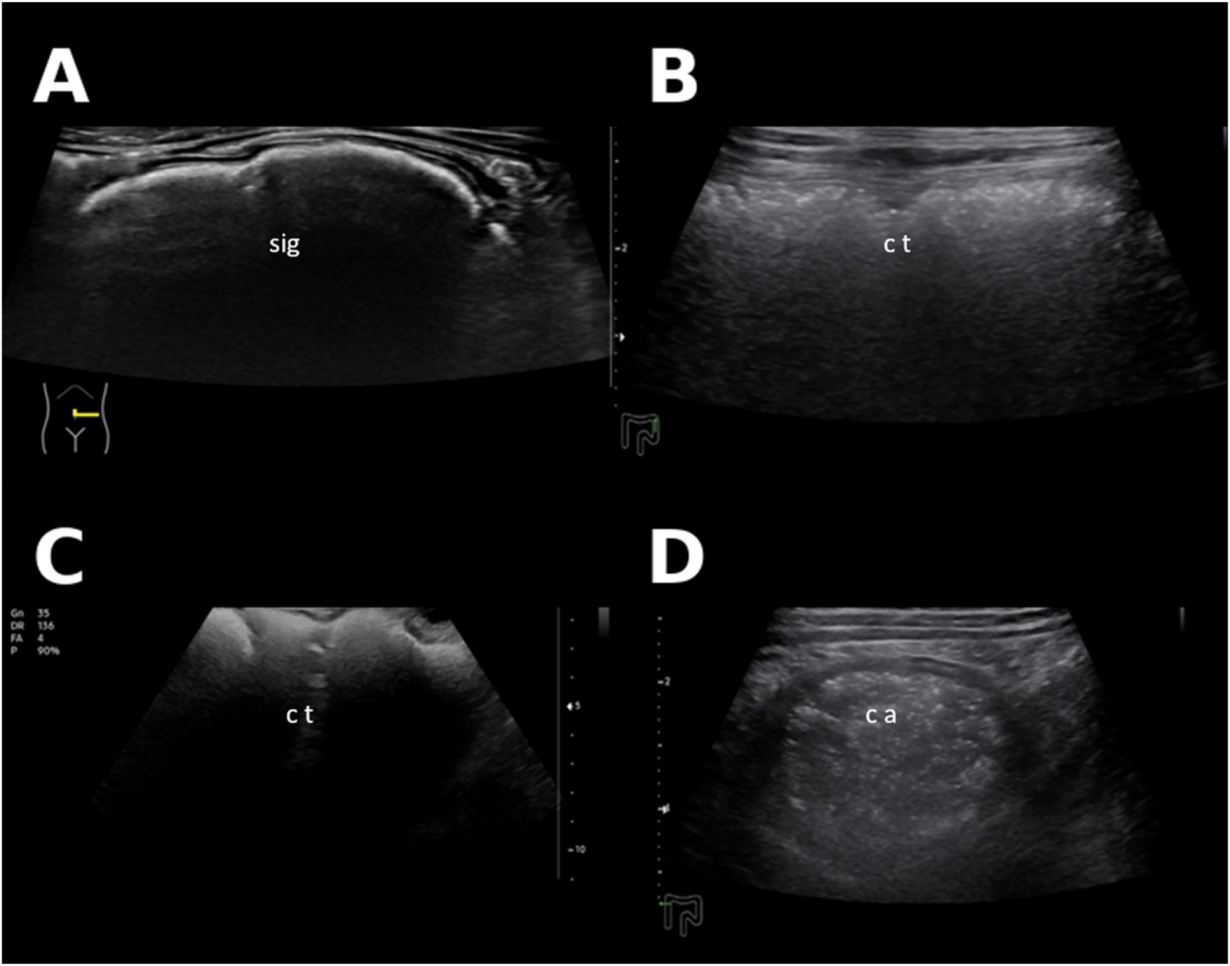
Representative transabdominal ultrasound appearances of colonic contents in children: **(A)** hard stool with strong anterior echoes and posterior shadowing; **(B)** soft stool with weak echoes and reduced shadowing; **(C)** intraluminal gas with reverberation artifacts; **(D)** liquid stool with a mixed “mousse-like” pattern (sig: sigmoid colon; ct: transverse colon; ca: ascending colon).

The Expert Position Statement (24) also provides at the following consensus on the US assessment of luminal contents. Solid stool within the colon appears as echogenic foci or a crescent-shaped echogenic area within the lumen, often accompanied by posterior acoustic shadowing. Liquid stool is typically anechoic on intestinal ultrasound (IUS) (agreement score = 0.77, “appropriate”). Intraluminal gas appears as highly echogenic foci. Multiple echogenic foci within grey shadowing indicate colonic gas trapped in solid stool, whereas multiple echogenic foci within anechoic regions represent gas bubbles in liquid stool. Figure 7 presents representative transabdominal US appearances of colonic content.

## 5 Constipation connected with slow colonic transit

Data on the pediatric population are limited, and current evidence is largely based on adult studies. Slow intestinal transit is a recognized mechanism underlying constipation, associated with impaired intestinal motility and prolonged colonic transit time (CTT) (18, 34).

**Figure 8:**
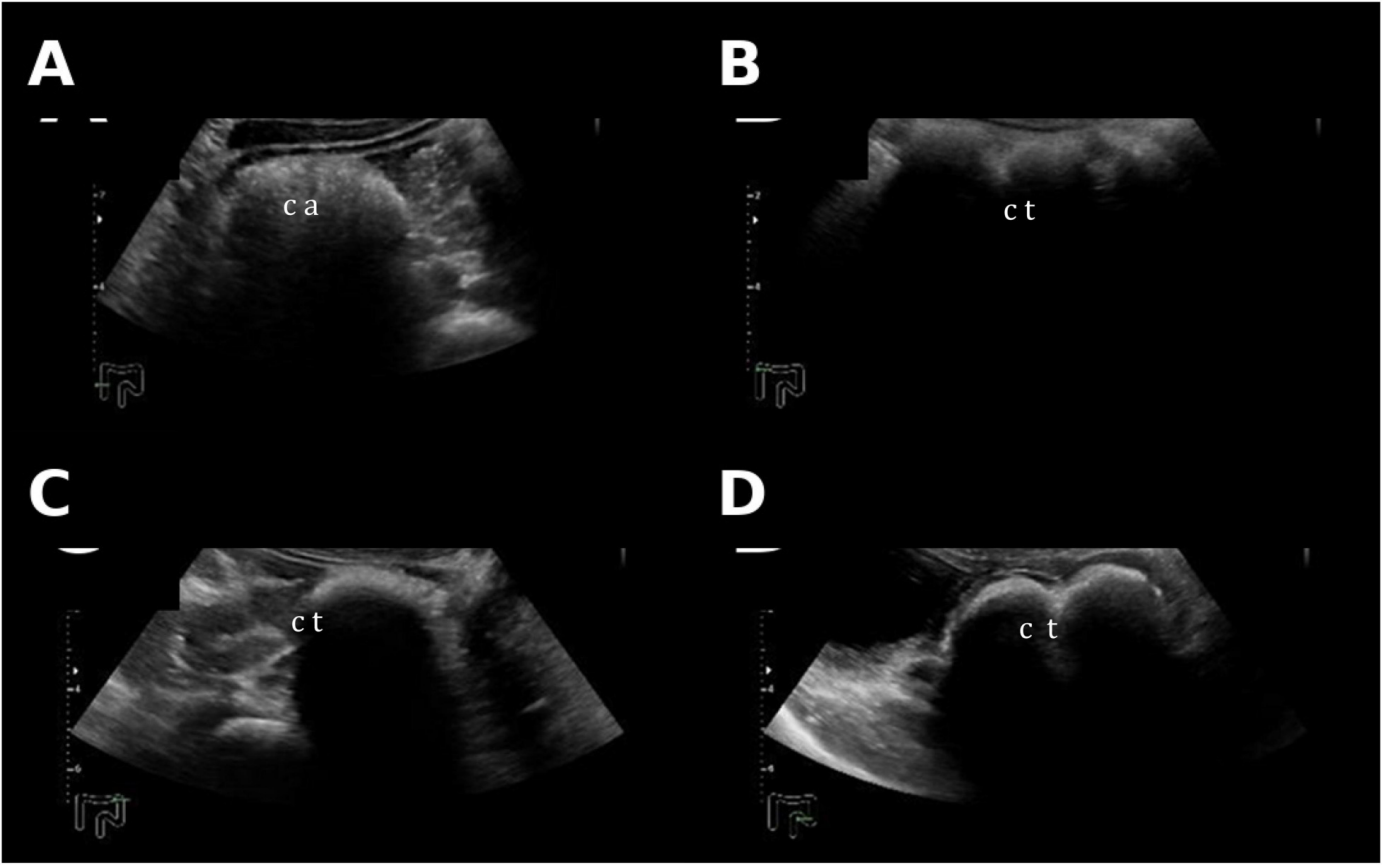
**(A–D)** severe fecal retention throughout the colon in a 12-year-old girl with slow colonic transit (c t: transverse colon, c a: ascending colon).

**Figure 9:**
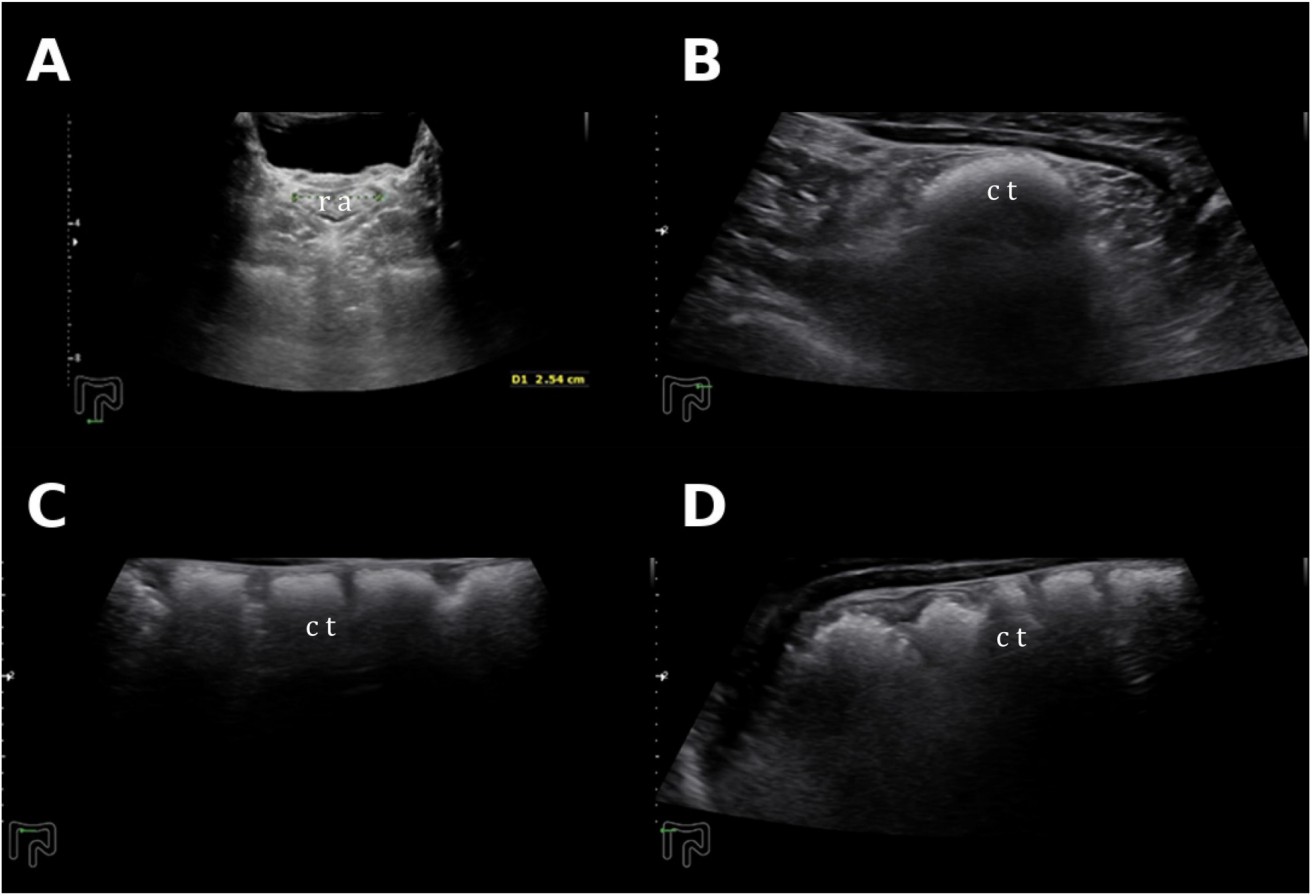
**(A–D)** severe fecal retention throughout the colon in a 14-year-old girl with slow colonic transit (c t: c transverse colon; ca: ascending colon; r a : rectum).

In children, US findings, including fecal impaction and colonic overfilling, were associated with significantly prolonged CTT, particularly in the left colon (17). This suggests that the US may provide indirect information on intestinal transit in children with FC. Similarly, studies in adults have shown that the US assessment of stool and gas distribution correlates with colonic transit patterns and treatment response (35). Although these findings cannot be directly extrapolated to children, they support the potential role of US as a non-invasive tool for evaluating intestinal motility. Figures 8,9 show severe fecal retention in a child with CTT. Detailed characteristics of the included studies are summarized in Table 3.

**Table 3:** A summary of available original articles addressing the assessment of ultrasound and colonic transit in constipation.

Study	Study population	Methods	Ultrasound parameters	Results	Conclusion
(17)	120 children with constipation (mean age 6.25 years) vs. 105 controls	US + digital rectal exam + CTT (radiopaque markers)	Rectal diameter, rectal filling, splenic flexure and transverse colon filling	Fecal impaction associated with prolonged total and left colonic transit time (*p* < 0.01); splenic flexure overfilling correlated with prolonged left CTT	US may reflect delayed colonic transit and stool retention in children
(35)	223 adults with chronic constipation (mean age 62.9 years)	US assessment of colon + treatment response analysis	Colon diameters, constipation index (CI), left/right (L/R) ratio	Higher CI associated with poor response to osmotic/fiber therapy; L/R ratio correlated with response to stimulant laxatives	US may help assess stool distribution and guide treatment; association with colonic transit patterns

## 6 Organic causes of constipation

Only about 5% of constipation cases in children and adolescents are attributable to an underlying organic cause, defined as recognizable anatomical, neurological, metabolic, or systemic disorders that directly impair intestinal motility or obstruct fecal transit, in contrast to FC. These may include Hirschsprung's disease, inflammatory bowel diseases, cystic fibrosis, Down syndrome, anorectal malformations, neuromuscular disorders, spinal cord abnormalities, celiac disease, and tumours located within the intestines. Some alarm signs may indicate a possible organic etiology. These include delayed passage of meconium (>48 h after birth), symptom onset before the age of 1 month, ribbon-like stools, absence of anal or cremasteric reflexes, hematochezia without anal fissures, growth failure or weight loss, abdominal distension, bilious vomiting, abnormal anal position, presence of a spinal hair tuft, deep sacral dimple, and changes in strength, tone, or reflexes of the lower extremities (13, 15).

### 6.1 Ultrasound findings in hirschsprung's disease

Congenital aganglionic megacolon, also named Hirschsprung's disease (HSCR), is the most common congenital gastrointestinal motility disorder (36, 37). It results from the absence of ganglion cells in the myenteric and submucosal plexuses of the distal bowel due to abnormal development of the enteric nervous system (37, 38). The clinical presentation is nonspecific and includes chronic constipation and intestinal obstruction (37). The diagnosis is based on rectal biopsy, anorectal manometry and imaging tests (39). To minimise radiation exposure, US has been proposed as an alternative method. It is as effective as a gastrografin enema in detecting HSCR (*p* = 1) (40). Also, hydrocolonic sonography—transabdominal US after a warm saline enema identified typical features of HSCR: a dilated segment, a distinct transition zone, a narrowed distal segment, a luminal diameter ratio >1.51, increased perfusion of the proximal bowel, and bowel wall thickening ≥1.9 mm (41).

The transverse RD is another parameter increased in HSCR (9, 42). Using a 3.5 MHz curved-array transducer placed above the symphysis, the maximal transverse RD was measured at a ≥15° caudal angle (9, 42). Lindert et al. reported a mean transverse RD of 3.45 cm in HSCR vs. 2.05 cm in healthy children, while patients with FC had significantly larger RD values (mean 4.36 cm; *p* = 0.001) (42).

Ultra-high-frequency US (UHFUS; 20–70 MHz) has also been used to distinguish ganglionic from aganglionic bowel. *Ex vivo* studies with 50 MHz probes showed that ganglionic segments have a thicker muscularis interna and a higher muscularis interna-to-muscularis externa ratio, consistent with histoimmunological findings (43, 44).

Although the literature is limited, available studies indicate that US may support the diagnosis of HSCR and help differentiate it from other causes of chronic constipation (40). Further *in vivo* research is needed.

### 6.2 Pediatric tumours presenting as constipation

#### 6.2.1 Ultrasound as a diagnostic tool

Ultrasonography is a first-line diagnostic modality for evaluating cancers in children. It allows for non-invasive assessment of the tumor's anatomical origin and boundaries without sedation or ionising radiation. This method also enables structural evaluation of the tumor based on characteristics such as solid, cystic, or vascular fragments. Abdominal US also allows assessment of metastases in adjacent organs (45). A recent retrospective study by aLucena at al (46). evaluated the effectiveness of single-phase computed tomography and US in the preoperative evaluation of solid abdominal tumors and their relationship to structures such as solid organs, vessels, gastrointestinal organs, and neuromuscular structures. Over the eight-year study period, 50 abdominal tumors were identified, and 44 (88%) were resected entirely with negative margins. In their analysis, they found that the comparison between single-phase CT and US US can be a good complement to single-phase CT in the preoperative evaluation of children with abdominal tumors.

#### 6.2.2 Tumors mimicking enlarged ampulla recti

Tumors arising in the retrorectal (presacral) space are rare lesions located between the rectum and sacrum, and include a heterogeneous group of congenital, neurogenic, osseous and miscellaneous neoplasms. Their estimated incidence is very low, and most series comprise only a few dozen cases from a single tertiary centre (47). Because of this rarity and the deep pelvic location, presacral tumors pose a well-recognised diagnostic challenge.

From an anatomical perspective, a presacral mass located directly behind the rectum can push the rectal wall forward, creating the impression of a “full rectum” on digital examination. If the mucosa feels smooth and compressible, the finding may be mistaken for intraluminal stool rather than an external mass, especially when suspicion is low. Diagnostic confusion increases when true fecal loading is present above the lesion, reinforcing the impression of rectal distension. For this reason, reviews on presacral tumors and tailgut cysts recommend careful evaluation of mucosal mobility and firmness during digital rectal examination and maintaining a low threshold for imaging when findings appear atypical or inconsistent with simple constipation (47).

**Figure 10:**
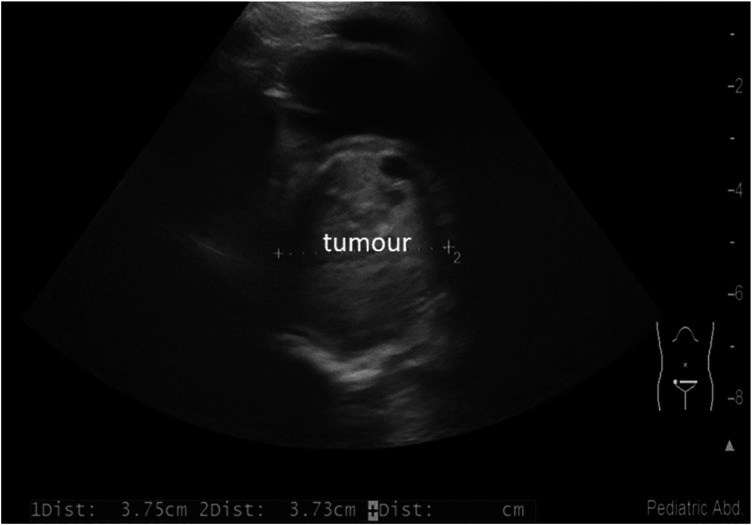
Tumour mimicking a distended rectal ampulla that appeared to be a yolk sac tumour in a 5-year-old girl with chronic constipation.

The pelvic masses in the presacral/retrorectal space can closely mimic a distended rectal ampulla or fecal loading, both clinically and on basic imaging (Figure 10). Rarity of these tumors, nonspecific symptomatology, subtle findings on digital rectal examination, and limitations of first-line imaging all contribute to delayed or incorrect diagnosis. Instead of classic alarm symptoms, patients typically present with chronic constipation, incomplete defecation, diffuse pelvic or sacral pain or discomfort, or a poorly localized sense of rectal pressure (47).

On plain radiographs or basic abdominal US, a retrorectal lesion may manifest as a posterior pelvic opacity or as apparent rectal or sigmoid distension, which can be attributed to fecal loading—particularly in patients referred for constipation. In contrast, contrast-enhanced CT and especially pelvic MRI allow precise localization of the lesion relative to the rectal wall and sacrum, characterization of cystic vs. solid components, and delineation of fat planes, thereby distinguishing a pelvic mass compressing the rectum from accurate intraluminal content (48).

Singer et al. reported a series of seven patients with retrorectal cysts that were initially misdiagnosed and treated as a variety of unrelated conditions, including fistulae, pilonidal cysts, perianal abscesses, psychogenic pain, lower back pain, post-traumatic or postpartum pain, and proctalgia fugax (49).

In a retrospective analysis of 112 patients with presacral tumors, Chen et al. (50) reported a high misdiagnosis rate of 29.5%, with lesions often initially mistaken for benign anorectal conditions such as hemorrhoids, constipation, abscess, or fistula. Tailgut cysts are particularly prone to mimic common pelvic and anorectal disorders (51). As shown by Johnson et al. (51), ultrasonography may be insufficient, and accurate diagnosis typically requires cross-sectional imaging, especially MRI. While ultrasonography is widely used to assess fecal loading and intestinal content, it has limited specificity for distinguishing neoplastic lesions from dense stool masses on standard imaging. Doppler evaluation and contrast-enhanced techniques may increase suspicion of a solid lesion by demonstrating abnormal vascularity, but definitive differentiation generally requires cross-sectional imaging (MRI/CT) or histopathological confirmation.

### 6.3 Ultrasound findings of constipation in children with neurological disorders

Constipation is highly prevalent in children with neurological impairment, particularly in those with cerebral palsy [CP], spina bifida, spinal dysraphism, neuromuscular disorders, and global developmental delay due to impaired gastrointestinal motility, reduced mobility, abnormal pelvic floor coordination, and communication difficulties. According to Alamdaran et al. (52), the prevalence of spina bifida and caudal regression in children with constipation was significantly higher compared with the healthy control group.

Clinical evaluation in this population is often challenging. (DRE) may be difficult to perform, uncomfortable, or poorly tolerated, and ultrasonography offers a valuable non-invasive alternative (53). Among this group of children, the most common disorder is cerebral palsy—a non-progressive neurological disorder resulting from injury in the developing brain of a fetus or infant and is one of the most common neurological diseases in children, affecting approximately 2–2.5 per 1,000 births (54–58). Children with CP are at increased risk of constipation, with reported prevalence rates ranging from 26%–74% (46, 56). They pass stool an average of once per week or once every 10 days, and these stools are hard and difficult to pass (54, 59). The causes of constipation in children with CP can be related to increased muscle tone, immobilization or hypomobility, mental retardation, poor diet with reduced intake of fibre and liquids, malnutrition, decreased colonic motility, changes in central nervous modulation control of the bowel, and drug adverse effects (anti-epileptic drugs) (54–56, 60).

**Figure 11:**
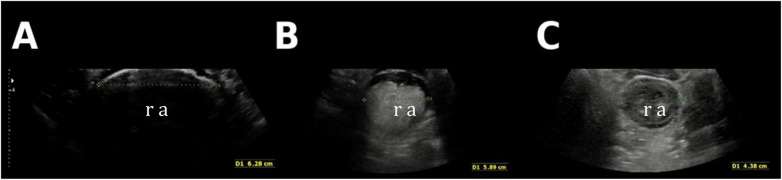
A 12-year-old girl with cerebral palsy and chronic constipation. Serial transabdominal ultrasound demonstrates persistent rectal dilatation despite treatment: **(A)** initial examination with a transverse rectal diameter of 6.28 cm, **(B)** follow-up examination after treatment showing a transverse diameter of 5.89 cm, and **(C)** subsequent follow-up with a transverse diameter of 4.38 cm. r a: rectal amulla.

Available evidence indicates that children with CP frequently develop neurogenic bowel dysfunction associated with chronic fecal retention and rectal dilatation. Studies focusing on neurogenic bowel populations, including children with CPand other neurological impairments, demonstrate that although RD may decrease with bowel management, complete normalization to values observed in healthy children is uncommon. Persistent rectal dilatation has been reported despite prolonged treatment and often necessitates long-term, individualized management strategies. These findings support the need for extended follow-up and objective monitoring of rectal size in this population (61, 62). Figure 11 presents a persistent enlarged RD in a girl with CP.

### 6.4 Ultrasound in children with anorectal disorders

#### 6.4.1 Endoanal ultrasound (EAUS)

EAUS is an established, minimally invasive imaging technique that provides precise visualization of the anal sphincter complex and adjacent structures, with three-dimensional reconstruction capabilities enhancing diagnostic clarity. Felt-Bersma (63) emphasizes its particular utility in benign anorectal pathologies, including cases presenting with constipation due to structural sphincter abnormalities.

EAUS reliably depicts both internal and external anal sphincter defects and can assess sphincter atrophy—a condition potentially contributing to obstructed defecation and impaired rectal evacuation. Moreover, EAUS demonstrates diagnostic performance comparable to MRI for sphincter evaluation, while offering advantages in terms of ease of use, patient tolerability, lower cost, and immediate accessibility. Although clinical indications have traditionally focused on fecal incontinence and perianal fistulae, the technique's ability to reveal subtle sphincter dysfunction supports its expanded application in children experiencing constipation secondary to anorectal dysfunction. Thus, EAUS serves as a valuable diagnostic tool in clarifying anatomical etiologies of constipation and guiding appropriate management interventions. In addition, EAUS is easy to perform, requires minimal training to master, and is generally well tolerated by patients (64).

Three-dimensional EAUS (3D-EAUS) plays an essential role in the anatomical assessment of anorectal structures in children with constipation secondary to anorectal pathology, particularly after surgical repair of anorectal malformations (ARM). In the study by Caldaro et al. (64), 3D-EAUS enabled detailed visualization of the internal anal sphincter (IAS) and external anal sphincter (EAS), revealing structural abnormalities such as IAS discontinuity or absence—predominantly in high-type ARM—and focal or diffuse EAS scarring depending on malformation type. These findings correlated with functional deficits documented by anorectal manometry and with clinical severity of constipation and/or fecal incontinence. Importantly, US findings provided prognostic value: patients with IAS discontinuity but preserved resting pressure (>20 mmHg) responded favorably to biofeedback and/or laxative therapy, whereas the absence of IAS combined with low resting pressure (<20 mmHg) was associated with the need for daily rectal enemas. Thus, 3D-EAUS not only clarifies the structural basis of defecatory dysfunction in this patient group but also supports individualized treatment planning by integrating anatomical imaging with functional assessment (64).

#### 6.4.2 Transperineal ultrasound (TPUS)

The TPUS is performed with a convex probe and a high-frequency linear probe placed, preferably a microconvex probe, on the perineum in sagittal and transverse planes. This approach enables visualization of the anal canal, the distal rectum, and the puborectalis sling, as well as basic pelvic floor dynamics during contraction or straining (in cooperative children). Although pediatric data are limited compared with adults, US may be particularly useful for: identification of abscesses and fistulas in the perineal region that can cause constipation, suspected pelvic floor dyssynergia; constipation associated with fecal incontinence (soiling); postoperative evaluation of anorectal malformations; mapping of the anal canal; and assessing sphincter integrity (65).

### 6.5 Perineal fistulae and abscess causing constipation

**Figure 12:**
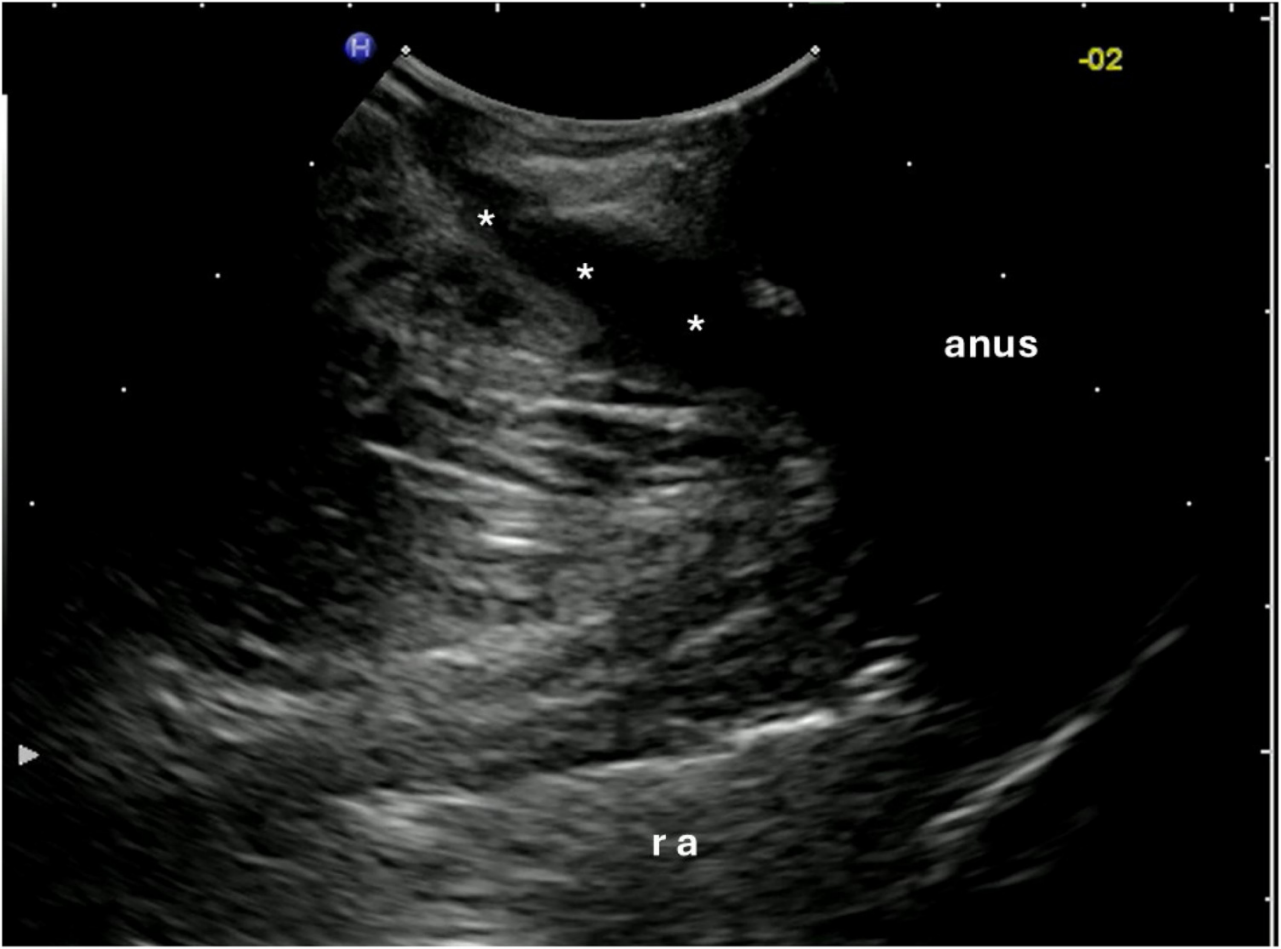
A hypoechoic tract (asterisks) originating from the posterior anorectal junction (6 o’clock position), extending through the trans-sphincteric space and reaching the skin (r a: rectal ampulla).

The fistula tract presents as a tube-shaped anechoic or hypoechoic area, and the internal opening can be identified after tracking the hypoechoic inward traverse of the perianal tissue. Meanwhile, the hypoechoic zone leading to the external opening on the perianal skin can be traced outward (Figure 12). A perianal abscess on US appears as a hypoechoic or anechoic fluid collection with irregular margins, usually avascular inside but with increased vascularity in the surrounding inflamed tissue**.**

Transperineal ultrasonography (TPUS) demonstrates high diagnostic accuracy for perianal disease across both adult and pediatric populations. In adults, it shows excellent sensitivity of 98% (95% CI 96%–100%) and positive predictive value of 95% (95% CI 90%–98%) for detecting perianal fistulae, identifying internal openings (the sensitivity of 91% (95% CI 84%–97%), classifying fistula tracts (the sensitivity of 92% (95% CI 85%–97%), and detecting abscesses (the sensitivity of 86% (95% CI 67%–99%) (65). In children, TPUS also performs strongly, with high sensitivity, specificity, and near-perfect agreement with surgical findings for assessing fistula complexity and internal openings (66). Additionally, it is well-suited for repeated follow-up due to its non-invasive nature (67) and has shown good to excellent agreement with MRI in evaluating treatment response, supporting its role as a reliable adjunct imaging modality, particularly when initial findings are consistent with MRI (68).

#### 6.5.1 Solitary rectal ulcer syndrome (SRUS)

**Figure 13:**
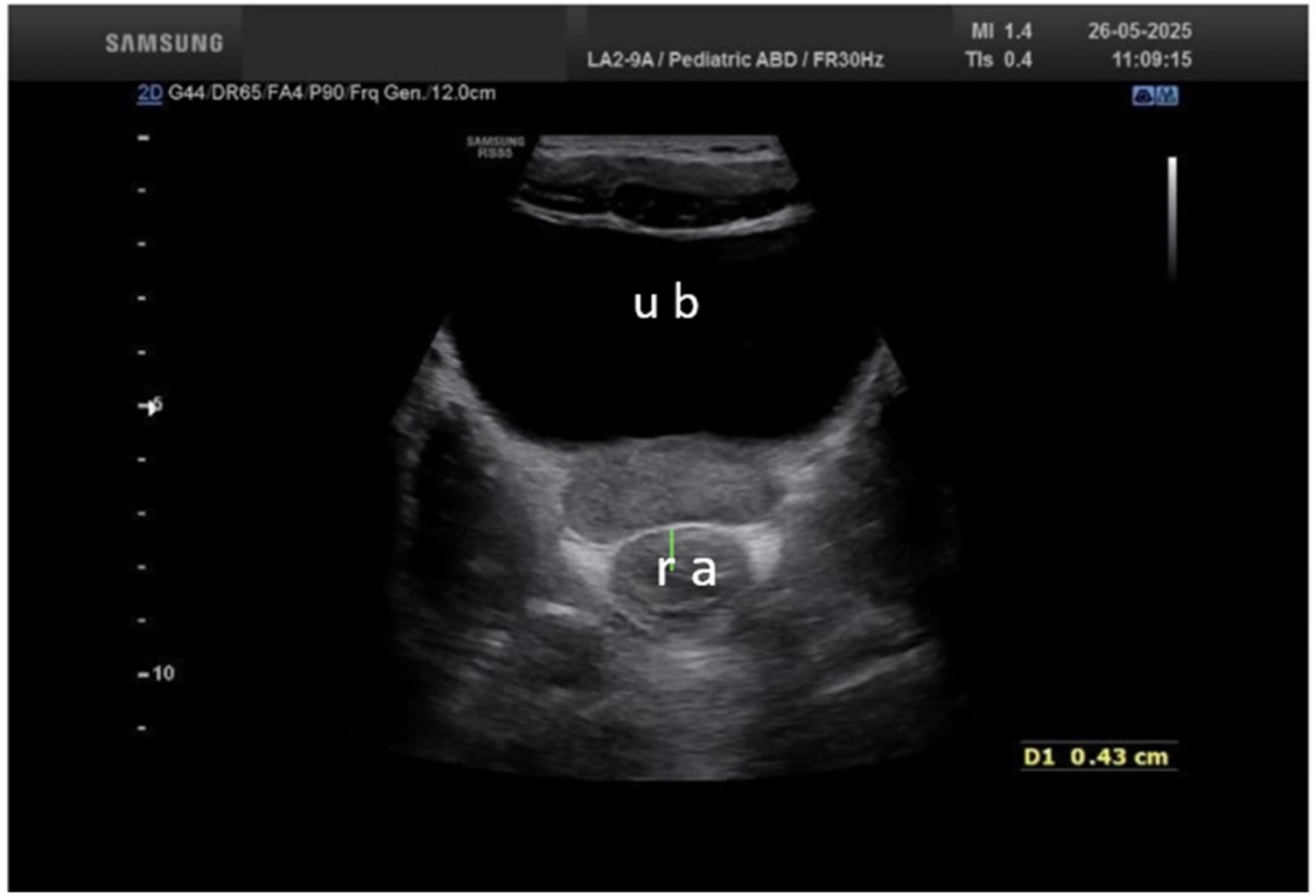
Thickened rectal wall in a 17-year-old boy with a solitary ulcer (r a: rectal ampulla) (u b: urinary bladder).

Solitary rectal ulcer syndrome (SRUS) is an uncommon, chronic, and benign rectal disorder frequently associated with constipation, abnormal defecation patterns and excessive straining. US techniques, including TPUS and EAUS, can be valuable tools for assessing anal sphincter thickness. According to the available literature (69), SRUS may be accompanied by thickening of the rectal wall and the internal and external anal spincters, as well as hypertrophy of the submucosal layer (Figure 13).

There are some limitations to the results and publications cited in this narrative review that should be acknowledged. First, a lot of studies evaluating US for constipation have been conducted in adult populations, limiting the direct applicability of their findings to children. Pediatric-specific data remain relatively limited, and extrapolation from adult studies should be interpreted with caution.

Second, there is considerable heterogeneity among the included studies. Differences exist across patient populations (age groups, disease severity), study design, and clinical settings. In addition, variability in US techniques, including the type of transducer used, patient preparation (e.g., bladder filling), and measurement protocols, further complicates direct comparison between studies.

Furthermore, many studies included relatively small sample sizes and were conducted in single-center settings, which may introduce selection bias and reduce the robustness of conclusions.

Particular attention was paid to potential sources of bias and heterogeneity, including: i) variability in age groups (pediatric vs. adult populations); ii) differences in ultrasound techniques and measurement protocols; iii) lack of standardized diagnostic thresholds. The included studies demonstrated substantial heterogeneity in terms of study design, patient populations, diagnostic methods, and outcome definitions. This represents an important limitation of the present review and necessitates careful consideration of potential sources of bias, as well as a critical interpretation of the findings.

Finally, due to the heterogeneity of study designs and outcome measures, a quantitative meta-analysis was not feasible, and the conclusions of this review are based on a narrative synthesis of the available evidence. This narrative review was undertaken as an initial attempt to synthesize the most recent guidelines and publications. Conducting a systematic review and, where possible, a meta-analysis would allow identification of specific knowledge gaps.

Nevertheless, even with this limited, cautiously interpreted data, the US remains a promising, non-invasive tool for evaluating constipation in children. However, further well-designed, multicenter studies with standardized protocols are needed to better define its role in clinical practice.

## 7 Conclusion

The US has become an increasingly important imaging modality for the evaluation and management of pediatric constipation, owing to its non-invasive nature, lack of ionizing radiation, and wide availability. Transabdominal intestinal US allows objective assessment of fecal loading through measurement of transverse RD, rectal wall thickness, and colonic filling. It also enables assessment of gastrointestinal luminal content and may have potential utility in the evaluation of colonic transit, although further validation is required in children. Perianal and transperineal US further enhances the diagnostic value of sonography by visualizing the anal canal, distal rectum, and pelvic floor structures, providing complementary information when digital rectal examination or invasive functional tests are not feasible.

Beyond diagnosis, US is also valuable for monitoring therapeutic response, as serial examinations may demonstrate reductions in RD and stool burden, supporting treatment adjustment and adherence, especially in children with severe refractory constipation, where US could be valuable to monitor improvement of new therapies regardless of symptom behaviour (70, 71). In addition, by allowing direct visualization of stool retention, US may serve as an effective educational tool for parents, particularly in cases of “hidden” constipation.
